# Long non-coding RNAs for osteosarcoma in the mouse: a meta-analysis

**DOI:** 10.18632/oncotarget.20128

**Published:** 2017-08-10

**Authors:** Shaopu Hu, Junli Chang, Yimian Li, Wenyi Wang, Edward C. Zou, Mengyao Guo, Qi Shi, Yongjun Wang, Yanping Yang

**Affiliations:** ^1^ Longhua Hospital, Shanghai University of Traditional Chinese Medicine, Shanghai, 200032, China; ^2^ Key Laboratory of Theory and Therapy of Muscles and Bones, Ministry of Education, Shanghai, 200032, China; ^3^ Consulting Engagement Management, Cerner, Kansas City, MO 64117, USA; ^4^ School of Rehabilitation Science, Shanghai University of Traditional Chinese Medicine, Shanghai, 201203, China

**Keywords:** lncRNA, osteosarcoma, meta-analysis, mice

## Abstract

Osteosarcoma, one of the most common primary bone malignances, is a leading cause of cancer death among children and adolescents. Recently, growing studies have found that long non-coding RNAs (lncRNAs) can interfere with the expression of various genes, and participate in the occurrence and development of malignancies. The purpose of this study is to evaluate the potential functions of lncRNAs as diagnostic biomarkers and therapeutic targets for osteosarcoma in mice, thus to direct the strict design for the future preclinical experiments and clinical trials. We systematically searched PubMed, Web of Science, Embase, China Knowledge Resource Integrated Database, VIP, Chinese BioMedical and Wan Fang Database from their initiation date to June 20, 2017. Two researchers independently screened the literatures and withdrew the data, which used the tumor volume and tumor weight as the outcome measures. A total of 10 studies were included, and the results of this meta-analysis revealed that lncRNAs could serve as the diagnostic biomarkers and therapeutic targets for osteosarcoma; and progression of osteosarcoma in mice could be inhibited via rescuing the abnormally expressed lncRNAs. It is necessary to carry out more rigorous basic experiments before lncRNAs can be further investigated in the clinical trials and used in future clinical practices.

## INTRODUCTION

Osteosarcoma is one of the most common primary bone malignancies, which often arises in the distal femur, proximal tibia, and proximal humerus. The nearby normal bone tissues are always damaged in bone malignancies [1]. Its incidence approximately makes up 60% of all children malignant bone tumors, especially among the males. Osteosarcoma is a leading cause of cancer death among children and adolescents [1, 2].

In recent years, with the progression of neoadjuvant chemotherapy and surgical treatment, the survival rate of osteosarcoma patients has been improved. The 5-year survival rate of patients with localized osteosarcoma is up to 80%, while only 20% of those with metastatic or recurrent disease can survive more than 5 years [3, 4]. Therefore, it is of great significance to seek out more effective diagnostic biomarkers and therapeutic targets for clinical treatment.

It has been reported that only 2% of the human genome DNA can encode proteins, while non-coding RNAs make up a significant proportion of the human genome DNA [5]. Non-coding RNAs are divided into two classes according to their transcription length: small non-coding RNAs and long non-coding RNAs (lncRNAs). LncRNAs are a class of endogenous non-coding RNAs with a length of more than 200 nucleotides, which can promote or impede the development of osteosarcoma [6]. For example, LINC00161 can enhance cisplatin-induced apoptosis through regulation of the miR-645-IFIT2 pathway, and down-regulation of LINC00161 contributes to cisplatin-resistance in osteosarcoma cells [7]. LncRNA ZEB1-AS1 functions as an oncogene in osteosarcoma, the proliferation and migration of osteosarcoma can be inhibited through down-regulating ZEB1-AS1 expression [8]. However, studies regarding lncRNAs are still in the early stage, and therefore, further investigations are necessary to explore more unknown mechanism of lncRNAs.

The purpose of this article is to evaluate the potential functions of lncRNAs as diagnostic biomarkers and therapeutic targets for osteosarcoma according to the published literatures; and to investigate the methodological quality of current studies to direct the strict design of the future preclinical experiments and clinical trials.

## RESULTS

### Literature selection

**Figure 1:**
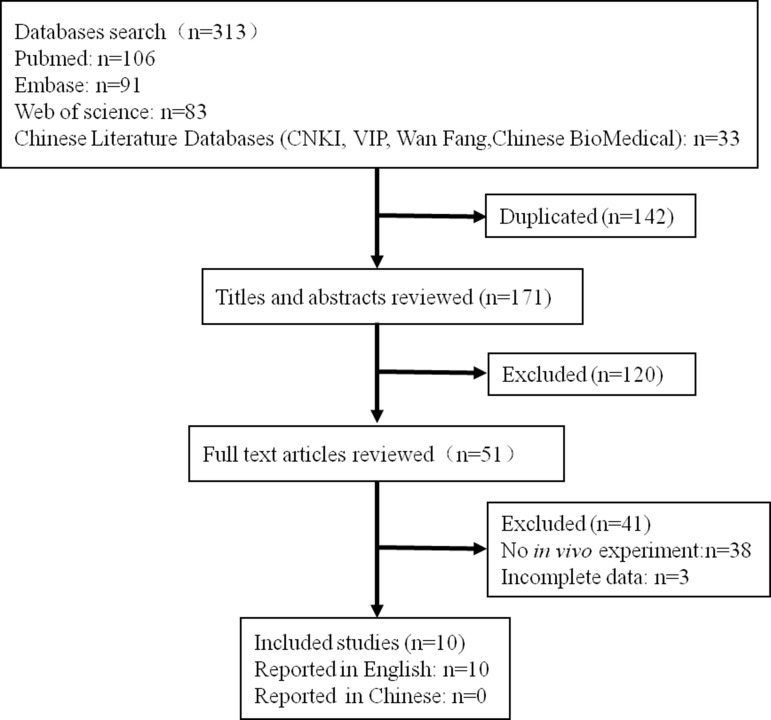
The flow diagram of the literature identification and selection process

The flow diagram of the literature identification and selection process is shown in Figure 1. We retrieved a total of 313 publications according to the search strategy described in the section of methods while 142 of the duplicated ones were excluded. After reviewing the titles and abstracts, 120 additional literatures were removed. After reading the full text of the 51 remaining publications, 41 of them were further excluded because of no *in vivo* experiments or incomplete data [9–11]. Ten of the literatures coincided with the inclusion criteria were included in this final meta-analysis. All included publications were reported in English [12–21].

### Study characteristics

Among all the 10 included studies, all of them used nude mice; 4 studies used female mice [12, 18–20], and 1 study used male mice [14], while the genders of mice in the 5 other studies were not reported.

The number of mice used in the 10 included studies were between 8 and 48. The detailed information of the feeding situations were not reported in the included studies. Among all the 10 included studies, 9 used subcutaneous injections to produce osteosarcoma xenograft models, one study used a peritoneal metastasis model [15].

In the included studies of this meta-analysis, the outcomes were represented as tumor weight, tumor volume, or both; diverse lncRNA types (HOTAIR, MALAT1, PVT1,TUG1, and so on) or functions (oncogenes or tumor suppressors) were reported; various osteosarcoma cell lines were used to produce osteosarcoma xenograft models (MG-63, U2 OS or MNNG/HOS cells) and different methods were used to produce xenograft models (subcutaneous inoculation or peritoneal metastasis) (Table 1).

**Table 1:** The characteristics of studies included in this meta-analysis

Studies	Characteristics of animals	Animal groups	Osteosarcoma xerograft methods	lncRNAs	Experimental groups	Control groups	Outcomes
Bo Wang 2015 [17]	15 BALB/c nude mice (6–8 weeks)	5/5/5	subcutaneous	HOTAIR	U2 OS+sh-HOTAIR	A:blank B:empty vector	Tumor volume Tumor weight
Menglin Cong 2016 [14]	10 male BALB/c nude mice	5/5	subcutaneous	TUSC7	MG-63+si-TUSC7	MG-63+NC	Tumor volume Tumor weight
Chu-Hai Xie 2016[18]	12 female athymic BALB/c nu/nu mice	6/6	subcutaneous	TUG1	U2 OS+si-TUG1	U2 OS+si-control	Tumor volume Tumor weight
Xianyi Cai 2015 [12]	12 female nude mice (4–5 weeks )	6/6	subcutaneous	MALAT1	MNNG/HOS+MALAT1 si-RNA	MNNG/HOS+non-specific si-RNA	Tumor volume Tumor weight
Fenyong Chen 2016 [13]	18 athymic BALB/c nude mice (4 weeks)	6/6/6	subcutaneous	BCAR4	MG-63+sh-BCAR4-1 MG-63+sh-BCAR4-2	MG-63+sh-control	Tumor volume Tumor weight
Quan Zhou 2016 [21]	8 nude mice	4/4	subcutaneous	PVT1	MG-63+Lv-sh-RNA-PVT1#1	MG-63+Lv-control	Tumor volume
Yongqiang Dong 2015 [15]	12 nude mice	6/6	peritoneal metastasis model	MALAT1	U2 OS+si-MALAT1	U2 OS+si-control	Tumor weight
Jiabing Sun 2016 [16]	12 nude mice	6/6	subcutaneous	FGFR3-AS1	MG-63+sh-FGFR3-AS1	MG-63+sh-Control	Tumor volume Tumor weight
Kaishan Ye 2017 [19]	20 female BALB/c athymic nude mice (4 weeks)	10/10	subcutaneous	GAS5	MG-63+Ad-GAS5	MG-63+Ad-NK	Tumor volume
Chun-Lin Zhang 2017 [20]	48 female BALB/c nude mice (4 weeks )	12/12/12/12	subcutaneous	FOXC2-AS1	MG-63+si-FOXC2-AS1 MG-63+FOXC2-AS1	MG-63+si-NC MG-63+FOXC2-AS1-NC	Tumor volume

Abbreviation: OS, osteosarcoma.

(NC = negative control).

### Quality evaluations of the included studies

Quality evaluation of each included study is shown in Table 2. As we can see in this diagram, no study in this meta-analysis has specifically described sample-size calculation, allocation concealment, blinded assessment of outcomes, or reported animals excluded from the analysis. Among all included studies, 5 studies reported inclusion and exclusion criteria, 2 studies reported randomization, 9 studies reported potential conflicts of interest and supported funding. Since there were only 10 published studies that met the inclusion criteria and the information of these studies were not comprehensive, the overall quality of the included literatures in this meta-analysis was low.

**Table 2:** Quality evaluation of the included studies

Studies	Sample-size calculation	Inclusion and exclusion criteria	Randomization	Allocation concealment	Reporting animals excluded from analysis	Blinded assessment of outcomes	Reporting potential conflicts of interest and study funding
Bo Wang 2015 [17]	No	Yes	Yes	No	No	No	No
Chu-Hai Xie 2016 [18]	No	No	No	No	No	No	Yes
Fenyong Chen 2016 [13]	No	Yes	No	No	No	No	Yes
Jiabing Sun 2016 [16]	No	No	No	No	No	No	Yes
Menglin Cong 2016 [14]	No	No	No	No	No	No	Yes
Quan zhou 2016 [21]	No	No	No	No	No	No	Yes
Xianyi Cai 2016 [12]	No	Yes	No	No	No	No	Yes
Yongqiang Dong 2015 [15]	No	No	No	No	No	No	Yes
Chun-Lin Zhang 2017 [20]	No	Yes	Yes	No	No	No	Yes
Kaishan Ye 2017 [19]	No	Yes	No	No	No	No	Yes

### The inhibitory effects of lncRNAs on the pathogenesis of osteosarcoma xenograft models (tumor volume and tumor weight) via correcting the abnormal expressed lncRNAs

Among all 10 included studies, diverse outcome measures (tumor weight, tumor volume, or both of them); diverse lncRNA types (HOTAIR, MALAT1, PVT1,TUG1, and so on) or functions (oncogenes or tumor suppressors) of lncRNAs; various osteosarcoma cell lines used to produce osteosarcoma xenograft models (MG-63, U2 OS or MNNG/HOS cells) and different methods of producing xenograft models (subcutaneous inoculation or peritoneal metastasis model) were all reported. These various factors could cause a high heterogeneity and in order to make the conclusion more convincible, we analyzed all included studies with various stratifications and used random-effects models to minimize the heterogeneity.

### All included studies that used tumor volume as the major outcome measure were stratified by the functions (oncogenes or tumor suppressors) of lncRNAs in the pathogenesis of osteosarcoma

**Figure 2:**
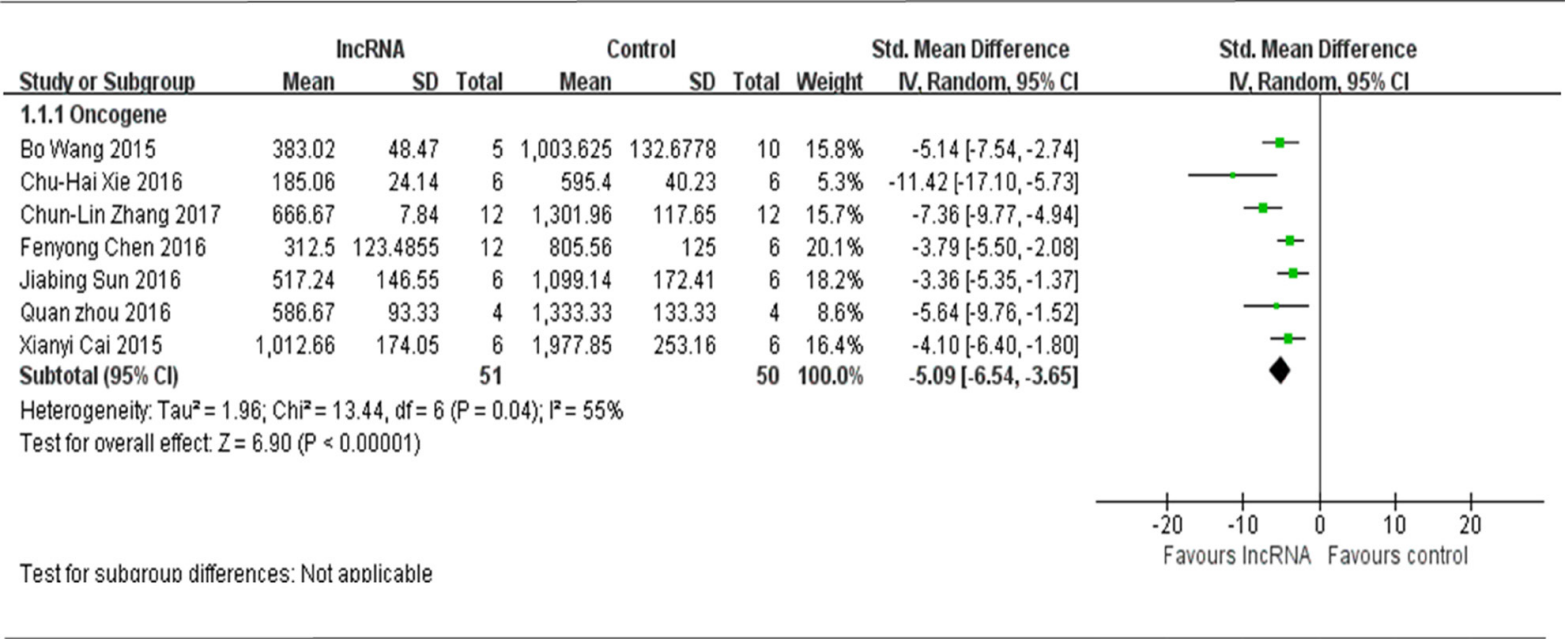
All included studies that used tumor volume as the major outcome measure were stratified by the functions of lncRNAs in the pathogenesis of osteosarcoma SD, standard deviation; CI, confidence interval.

There were 9 studies that used tumor volume as the major outcome measure in this meta-analysis, with 7 of them reporting lncRNAs function as the oncogenes. Therefore, all the data were extracted from these 7 studies and pooled for reanalysis [12, 13, 16–18, 20, 21]. There were a total of 51 mice in the experimental group and 50 mice in the control group. The results of the forest plot using the random-effects model suggested that down-regulation of tumor onco-lncRNAs suppressed the growth of osteosarcoma xenografts *in vivo.* The pooled MD = [−5.09]; 95% confidence interval [CI]: [−6.54]−[−3.65]; *p* < 0.00001(Figure 2).

The other 2 studies used tumor volume as the major outcome measure and lncRNAs function as the tumor suppressors. While the technique used to determine the function of the target lncRNA was different, one tumor suppressor was overexpressed, while another was silenced, therefore, the data from these 2 studies could not be pooled. One study reported that long non-coding RNA TUSC7 was down-regulated in osteosarcoma cells. Silence of TUSC7 in MG-63 promoted osteosarcoma growth in subcutaneous inoculation of MG-63+si-TUSC7 produced osteosarcoma xenograft models [14]. The other study showed that lncRNA GAS5 was down-regulated in osteosarcoma and its overexpression suppressed tumor growth of osteosarcoma [19].

### The above mentioned studies reported lncRNAs as oncogenes or tumor suppressors, used tumor volume as the major outcome measure, were stratified by the following factors

#### Osteosarcoma cell lines used to produce osteosarcoma xenograft models

**Figure 3:**
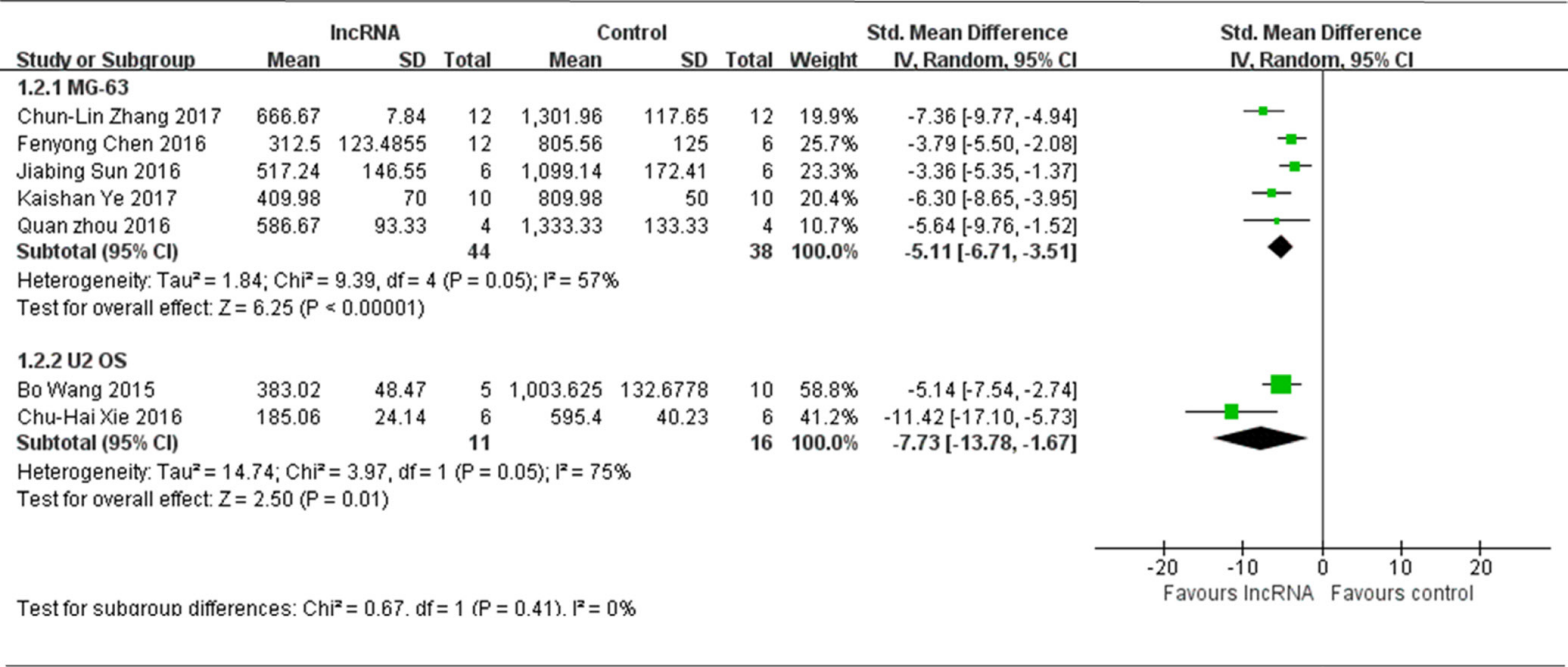
All studies that used tumor volume as the major outcome measure were stratified by osteosarcoma cell lines used to produce osteosarcoma xenograft models SD, standard deviation; CI, confidence interval.

Among all 9 studies that used tumor volume as the major outcome measure in this meta-analysis, 6 studies used MG-63 to produce osteosarcoma xenograft models, while one study was not pooled because that, compared to the control, the efficacy of lncRNA intervention was to promote tumor growth [14], therefore, all the data extracted from the other 5 studies, with decreased tumor growth after lncRNA intervention, were pooled for reanalysis [13, 16, 19–21]. There were a total of 44 mice in the experimental group and 38 mice in the control group. The results of the forest plot using the random-effects model suggested that tumor volumes were significantly decreased by correcting the aberrant expression of lncRNAs. The pooled MD = [−5.11]; 95% confidence interval [CI]: [−6.71]−[−3.51]; *p* < 0.00001(Figure 3, upper part).

Two studies used U2 OS to produce osteosarcoma xenograft models [17, 18]. There were a total of 11 mice in the experimental group and 16 mice in the control group. The results of the forest plot suggested that tumor volumes were significantly decreased by correcting the aberrant expression of lncRNAs. The pooled MD = [−7.73]; 95% confidence interval [CI]: [−13.78]−[−1.67]; *p* = 0.01(Figure 3, lower part).

Only 1 study used MNNG/HOS cell line to produce osteosarcoma xenograft models [12]. This study confirmed that the expression of MALAT1 was up-regulated in both human osteosarcoma cell lines and tissues, and knockdown of MALAT1 delayed the tumor growth *in vivo*.

By comprehensively analyzing the results in Figure 3, it can be speculated that the tumor volume was more significantly reduced when U2 OS was used to produce osteosarcoma xenograft models than MG-63.

#### Methods for producing xenograft models

**Figure 4:**
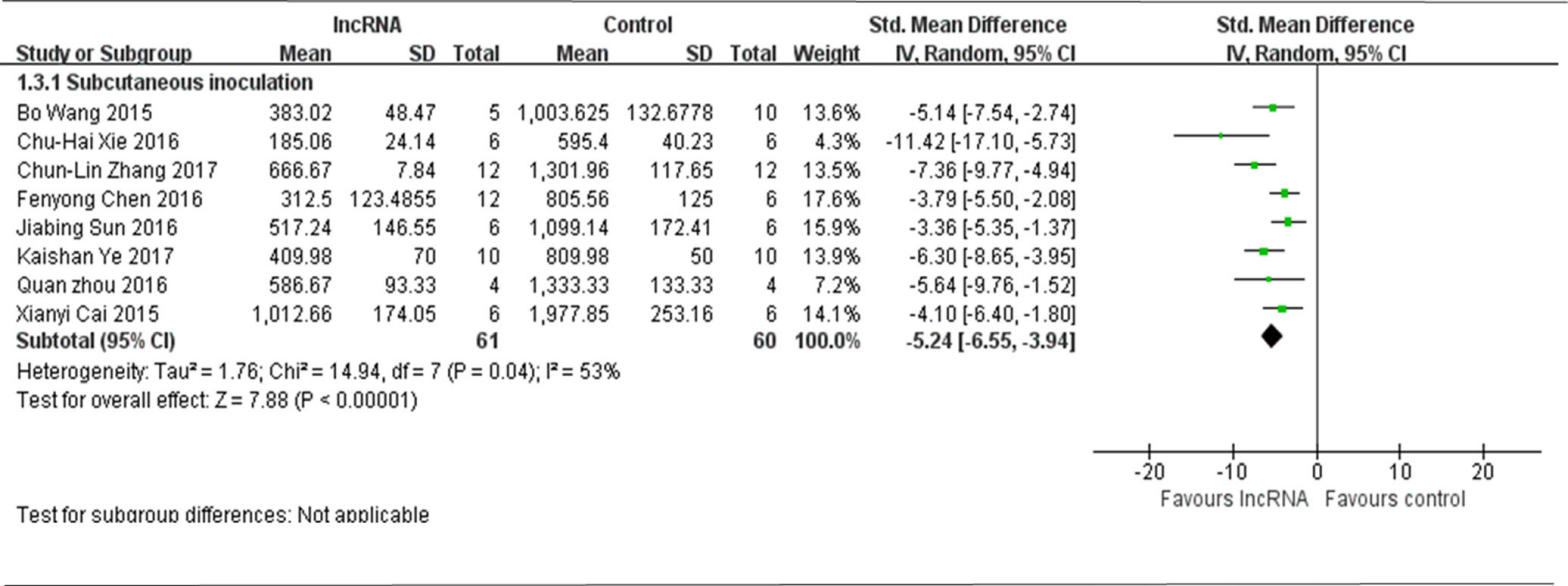
All studies that used tumor volume as the major outcome measure were stratified by injection sites of osteosarcoma cells SD, standard deviation; CI, confidence interval.

All 9 studies used tumor volume as the major outcome measure and subcutaneous inoculation to produce osteosarcoma xenograft models, while data from one study was not be pooled due to the efficacy of lncRNA intervention was to promote tumor growth versus the control [14]. Therefore, all the data extracted from the other 8 studies, with decreased tumor growth after lncRNA intervention, were pooled for reanalysis [12, 13, 16–21]. There were 61 mice in the experimental group and 60 mice in the control group. The results of the forest plot indicated that tumor volume was significantly decreased by correcting the aberrant expression of lncRNAs. The pooled MD = [−5.24]; 95% confidence interval [CI]: [−6.55]−[−3.94]; *p* < 0.00001(Figure 4).

### All included studies that used tumor weight as the major outcome measure were stratified by the functions (oncogenes or tumor suppressors) of lncRNAs in the pathogenesis of osteosarcoma

**Figure 5:**
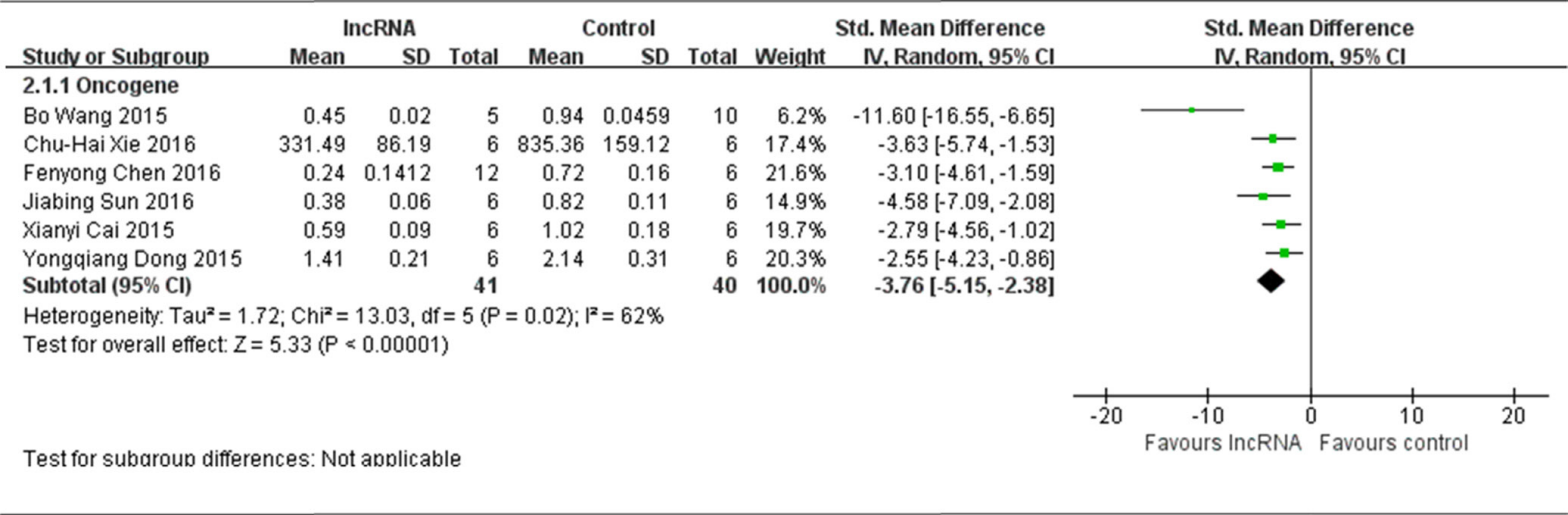
All included studies that used tumor weight as the major outcome measure were stratified by the functions of lncRNAs in the pathogenesis of osteosarcoma SD, standard deviation; CI, confidence interval.

There were 7 studies that used tumor weight as the major outcome measure in this meta-analysis. Among the 7 included studies, 6 reported lncRNAs function as the oncogenes. Therefore, all the data from the 6 studies were extracted and pooled for reanalysis [12, 13, 15–18]. There were a total of 41 mice in the experimental group and 40 mice in the control group. The results of the forest plot with the random-effects model indicated that down-regulation of tumor onco-lncRNAs suppressed the growth of osteosarcoma xenografts *in vivo.* The pooled MD = [−3.76]; 95% confidence interval [CI]: [−5.15] −[−2.38]; *p* < 0.00001(Figure 5).

Only 1 study reported lncRNA functions as the tumor suppressor and used tumor weight as the major outcome measure, therefore the data could not be pooled for reanalysis [14]. This study reported that silence of TUSC7 promoted osteosarcoma growth both in vitro and in vivo as mentioned above*.*

### The above mentioned studies reported lncRNAs as oncogenes or tumor suppressors used tumor weight as the major outcome measure, were stratified by the following factors

#### Osteosarcoma cell lines used to produce osteosarcoma xenograft models

**Figure 6:**
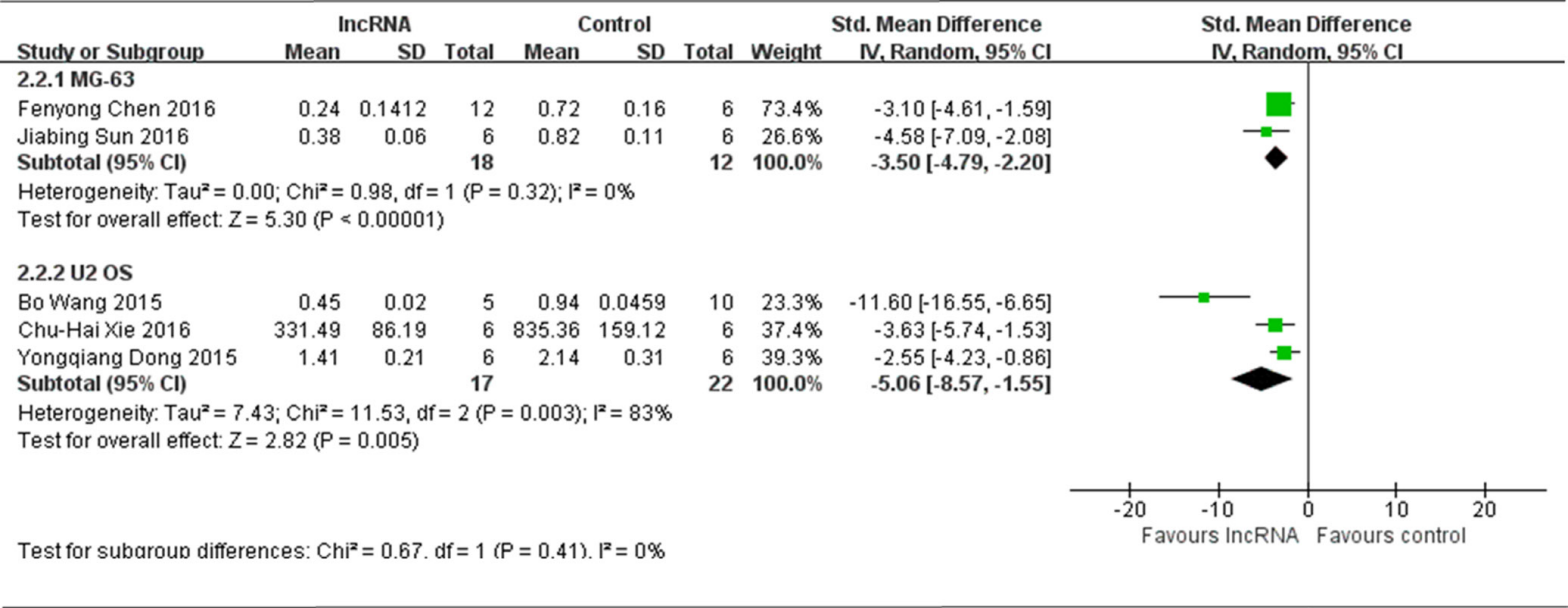
All studies that reported lncRNAs as tumor oncogenes and used tumor weight as the major outcome measure were stratified by osteosarcoma cell lines used to produce osteosarcoma xenograft models SD, standard deviation; CI, confidence interval.

Among that 6 studies reported lncRNAs function as tumor oncogenes and used tumor weight as the major outcome measure, only 2 studies used MG-63 to produce osteosarcoma xenograft models [13, 16]. According to the meta-analysis, there were a total of 18 mice in the experimental group and 12 mice in the control group. The results of the forest plot with the random-effects model suggested that the tumor weight was significantly decreased by correcting the aberrant expression of lncRNAs. The pooled MD = [−3.50]; 95% confidence interval [CI]: [−4.79]−[−2.20]; *p* < 0.00001 (Figure 6, upper part). Three studies used U2 OS to produce osteosarcoma xenograft models [15, 17, 18]. According to the meta-analysis, there were 17 mice in the experimental group and 22 mice in the control group. The results of the forest plot suggested that tumor weight was significantly decreased by correcting the aberrant expression of lncRNAs. The pooled MD = [−5.06]; 95% confidence interval [CI]: [−8.57]−[−1.55]; *p* = 0.005 (Figure 6, lower part). Only 1 study used MNNG/HOS cell line to produce osteosarcoma xenograft models [12], therefore the data could not be pooled for reevaluation. This study confirmed silenced MALAT1 inhibited osteosarcoma growth both *in vitro* and *in vivo* as mentioned above.

According to the comprehensive analysis of Figure 6, it can be speculated that the tumor weight was more significantly decreased when U2 OS was used to produce osteosarcoma xenograft model than MG-63.

#### Methods for producing xenograft models

**Figure 7:**
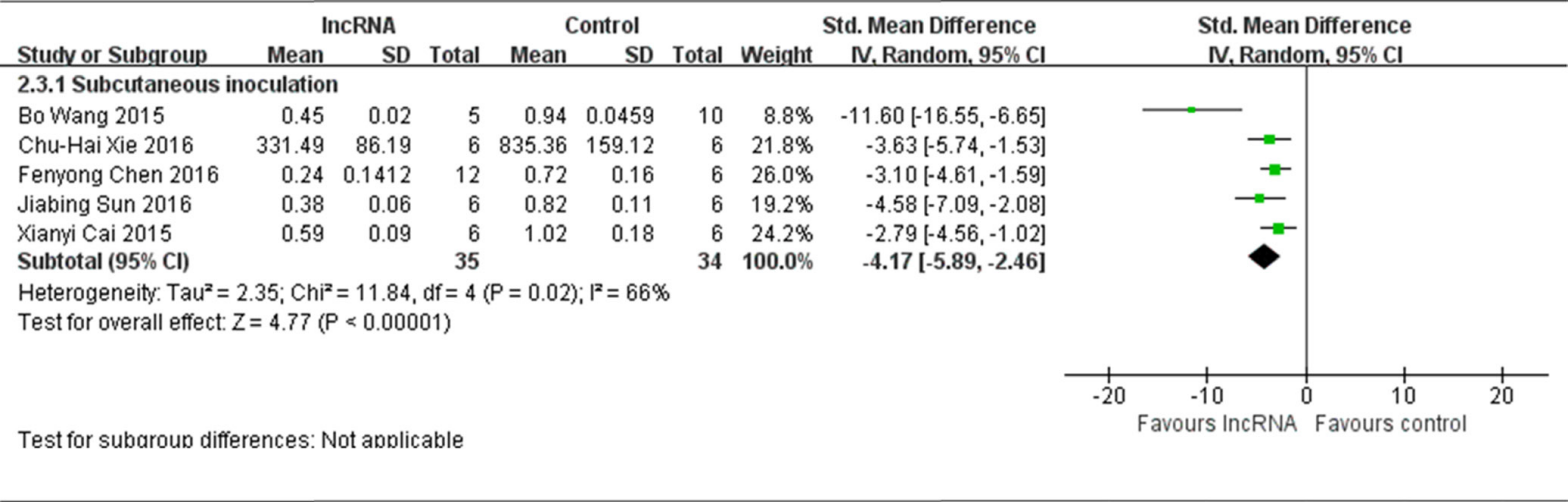
All studies that reported lncRNAs as tumor oncogenes and used tumor weight as the major outcome measure were stratified by injection sites of osteosarcoma cells SD, standard deviation; CI, confidence interval.

Among the 6 studies that reported lncRNAs function as tumor oncogenes and used tumor weight as the major outcome measure, 5 studies used subcutaneous inoculation to produce osteosarcoma xenograft models [12, 13, 16–18]. Therefore, data from these 5 studies were pooled for reanalysis. According to the meta-analysis, there were 35 mice in the experimental group and 34 mice in the control group. The results of the forest plot indicated that tumor weight was significantly decreased by correcting the aberrant expression of lncRNAs. The pooled MD = [−4.17]; 95% confidence interval [CI]: [−5.89]−[−2.46]; *p* < 0.00001 (Figure 7). Only 1 study used peritoneal metastasis model [15], therefore the data could not be pooled for reevaluation. This study reported that MALAT1 was up-regulated two folds in osteosarcoma tissues and a knockdown of MALAT1 could suppress the tumor growth via PI3K/AKT signaling pathway.

#### The names of lncRNAs

**Figure 8:**
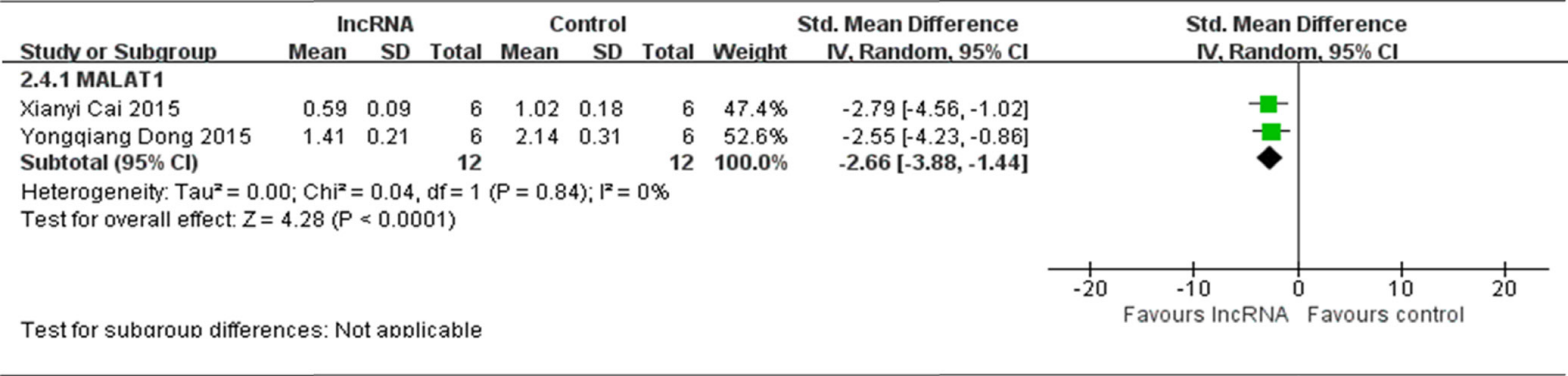
All included studies that reported lncRNAs function as tumor oncogenes and used tumor weight as the major outcome measure were stratified by the names of lncRNAs SD, standard deviation; CI, confidence interval.

The aim of this stratification is to explore the functions of different lncRNAs for osteosarcoma in the mice; therefore, the data about the same lncRNA in more than two included studies was pooled for reevaluation. Among the 6 studies that reported lncRNAs function as tumor oncogenes and used tumor weight as the major outcome measure, 5 different lncRNAs (HOTAIR, TUG1, BCAR4, MALAT1 and FGFR3-AS1) were reported. However only MALAT1 could be pooled for reevaluation due to the number of included studies [12, 15]. There were a total of 12 mice in the experimental group and 12 mice in the control group. The results of the forest plot suggested that tumor weight was significantly decreased by down-regulating tumor onco-lncRNA MALAT1 expression. The pooled MD = [−2.66]; 95% confidence interval [CI]: [−3.88]−[−1.44]; *p* < 0.0001(Figure 8). The other 4 lncRNAs were independently reported in 1 study[ 13, 16–18], and therefore the data could not be pooled.

### Begg’s funnel plot analysis

**Figure 9:**
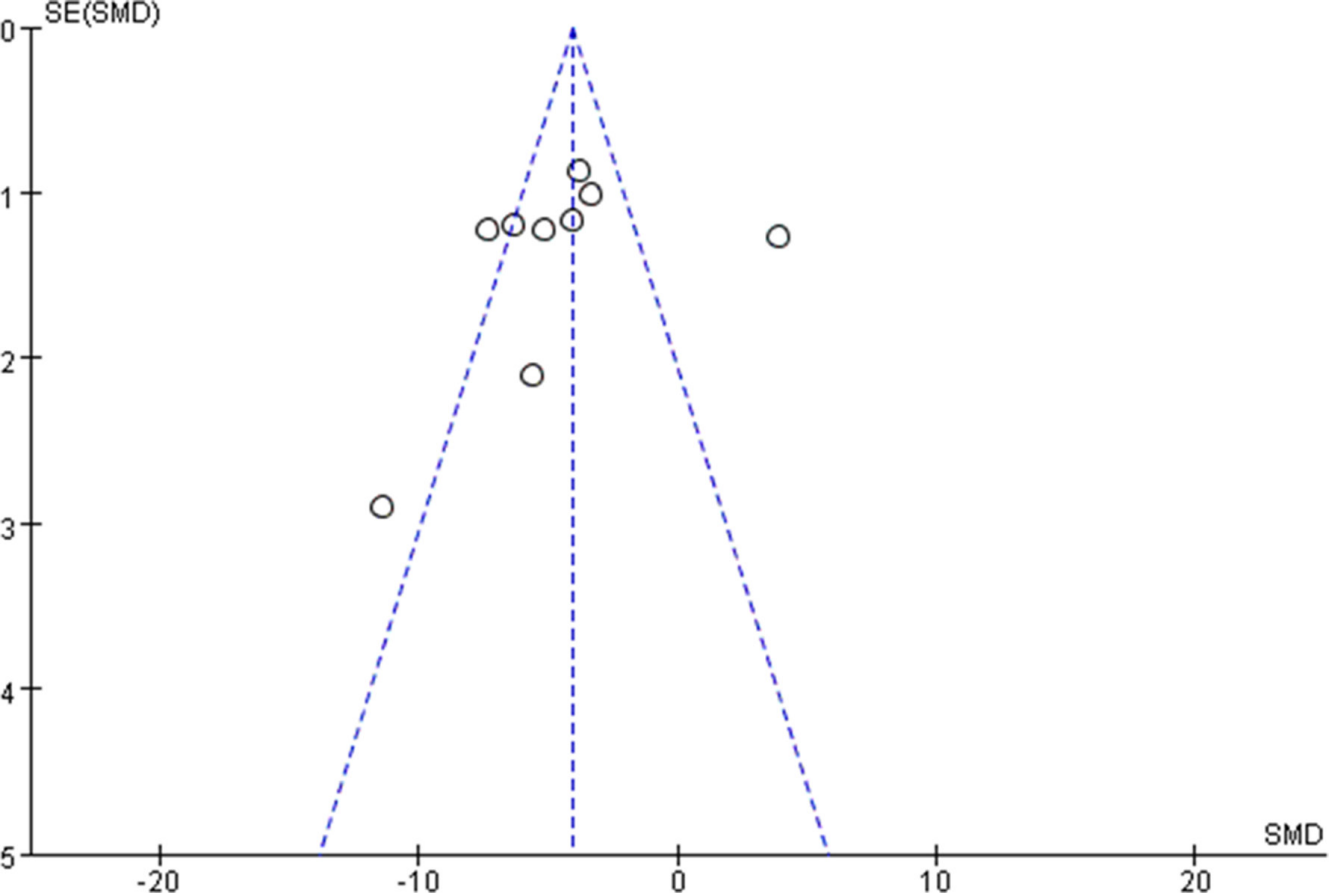
Funnel plot analysis to estimate publication bias for lncRNAs on tumor volume in the OS mice

**Figure 10:**
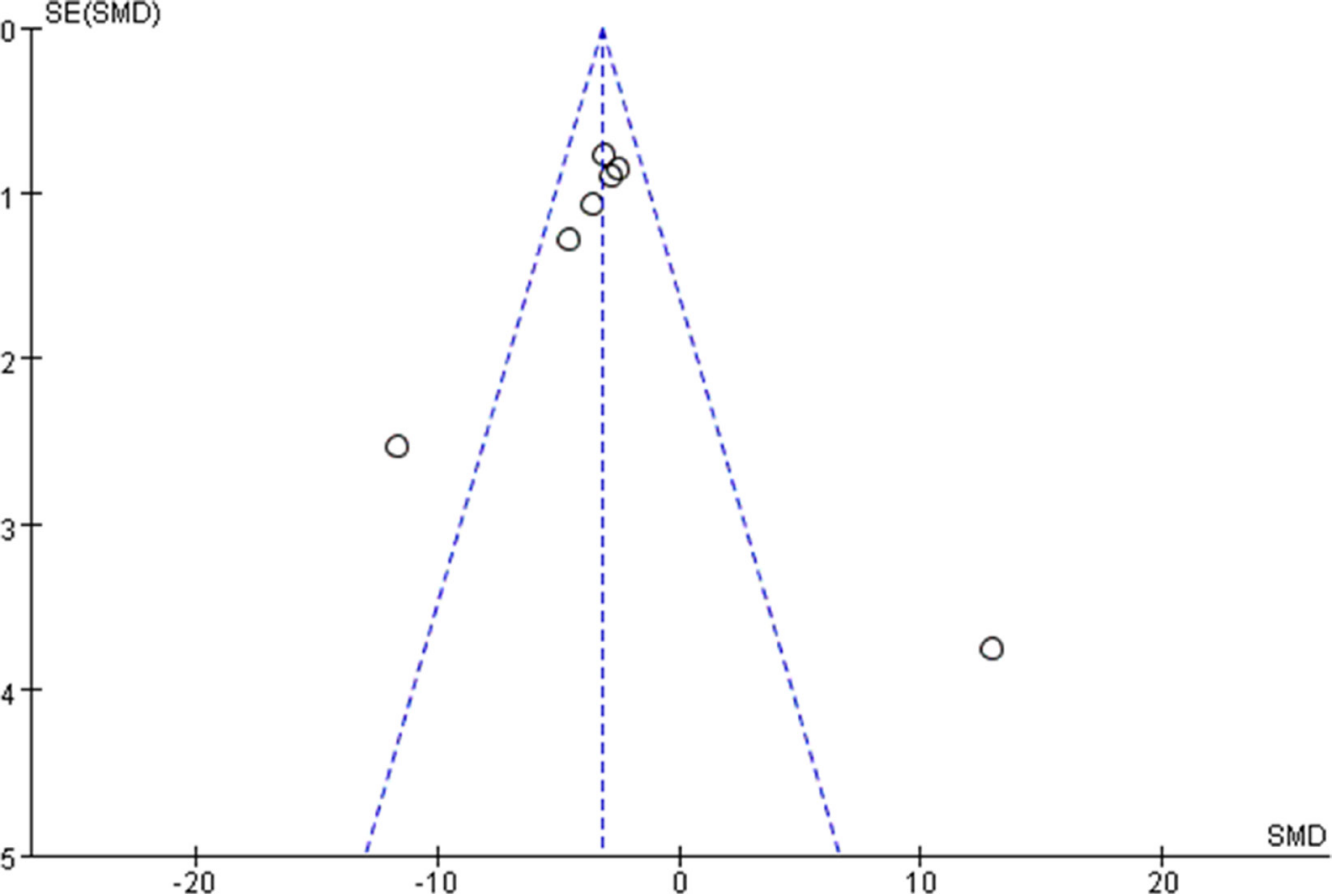
Funnel plot analysis to estimate publication bias for lncRNAs on tumor weight in the OS mice

To explore whether our findings were influenced by the potential publication bias, a Begg’s funnel plot was used for analysis. The results showed no any obvious asymmetry for either tumor volume(Figure 9) or tumor weight (Figure 10). Therefore, the conclusion of this meta-analysis was not influenced by publication bias.

## DISCUSSION

In the past decades, it was widely accepted that lncRNAs were transcriptional “noises” and the function mechanisms of lncRNAs in diseases were not well known [22]. LncRNAs, in fact, could up-regulate or down-regulate the expression of genes by stimulating or inhibiting RNA polymerase II recruitment, and then promote or inhibit the development of various diseases [23, 24]. Recent published studies on dysregulated lncRNA expressions in many cancer types reveal that lncRNAs either can function as tumor suppressors or tumor oncogenes and, therefore, could affect the development of malignancies [22, 25].

Accumulating reports of lncRNAs on osteosarcoma in the mouse models implied that many lncRNAs could promote the progression of tumor *in vivo,* while the tumor volume and tumor weight were significantly decreased by down-regulating the expressions of these lncRNAs. Therefore, these evidences indicated that lncRNAs may be used as therapeutic targets for osteosarcoma in future.

Nine different long non-coding RNAs were studied in these 10 included articles, with 7 lncRNAs including MALAT1 [12, 15], BCAR4 [13], FGFR3-AS1 [16], HOTAIR [17], TUG1 [18], FOXC2-AS1 [20] and PVT1 [21] were up-regulated in osteosarcoma cells or patients. Knockdown or down-regulation of these lncRNAs significantly inhibited cell proliferation *in vitro* and delayed tumor growth in osteosarcoma xenograft models. Therefore, it has been reported that these lncRNAs function as tumor oncogenes in osteosarcoma. Two different lncRNAs were down-regulated in osteosarcoma cells, the silence of TUSC7 [14] promoted tumor growth *in vivo*, and the overexpression of GAS5 [19] inhibited OS cell growth *in vitro* and *in vivo*. Therefore, it has been reported that these lncRNAs can act as tumor suppressors in OS.

Although a large number of studies have demonstrated that lncRNAs can be used as potential targets for clinical treatment of osteosarcoma, it remains unknown whether these lncRNAs can be directly used in clinical practice. There is a great deal of difference between animal experiments and clinical trials, while animal experiments being able to help us understand the mechanism of the disease and potentially detect the safety and efficacy of the new therapeutics. Therefore, animal experiments are an important basis for the implementation of clinical trials [26, 27]. Among various published animal studies, it is common for bias to exist due to the following factors: the breeding backgrounds of animals, the intervention methods, the outcome indicators and so on. Therefore, an accepted way is required to analyze all the existing data systematically. Meta-analysis could increase the correlation between animal models and clinical trials, and improve the defects of animal experiments [28, 29].

This is the first meta-analysis to systematically collect the data of animal studies and to evaluate the potential functions of lncRNAs as diagnostic biomarkers and therapeutic targets for osteosarcoma. We systematically searched the databases both in English and Chinese from their initiation date to June 20, 2017 to ensure the comprehensiveness of the retrieval. Screening the literatures, extracting the data and evaluating the methodological quality of included studies were executed by two researchers (S.P.H. and J.L.C.) independently. During the process, all divergences were decided by discussion with a third investigator (Y.P.Y.) to minimize the bias. In this meta-analysis, we systematically evaluated the quality of all included studies that reported the influences of lncRNAs on osteosarcoma in mice and reviewed the potential functions of lncRNAs as the therapeutic targets for osteosarcoma in future clinical practices.

All literatures and data included in this meta-analysis were collected strictly according to the inclusion/exclusion criteria aimed at improving the quality of included studies and evading the bias, though some inevitable factors still existed. As we could see in Tables 1 and 2, only five studies reported the genders of mice. No study in this meta-analysis has specifically described sample-size calculation and allocation concealment, blinded assessment of outcomes and reported animals excluded from analysis. Among all included studies, 5 studies reported inclusion and exclusion criteria, with 2 studies reported randomization. Therefore, the defects of low quality and high heterogeneity exist in this meta-analysis.

Heterogeneity is commonly unavoidable and generally acceptable among any meta-analysis including our current meta-analysis. To minimize the heterogeneity and improve the quality of evaluation, the most appropriate method should be adopted when different intervention methods are evaluated for different diseases. As we mentioned above, among all the 10 included studies, diverse outcome measures, and diverse lncRNA types or functions, various osteosarcoma cell lines used to produce osteosarcoma xenograft models and different methods for producing xenograft models were reported. These various elements caused high heterogeneities. In order to make the conclusion more convincing, we analyzed all included studies with various stratifications and used random-effects models to minimize the heterogeneities.

After systematically analyzing the stratifications reported above, we further evaluated the inhibitory influences of lncRNAs in the pathogenesis of osteosarcoma xenograft models via correcting the abnormally expressed lncRNAs. With comprehensive analysis of all data, our results demonstrated that, under the same conditions, the inhibitory effects on the tumor volume and tumor weight were better when U2 OS cell lines were used to produce osteosarcoma xenograft models. Though various factors in the subgroups resulted in some data that could not be pooled for reevaluation, our results indicated that the inhibitory influences of lncRNAs on tumor growth, by correcting the abnormally expressed lncRNAs. Therefore, this suggests that the effects of different interventions on osteosarcoma are specific, which provides a theoretical foundation for the future design of the animal experiments and clinical trails. However, due to the limited number and quality of included studies, more reliable experiments to prove this conclusion are required in the future.

In conclusion, the results of our meta-analysis suggest that lncRNAs are the potential diagnostic biomarkers and therapeutic targets for osteosarcoma. This will provide a theoretical basis for the future development of animal experiments and new therapeutic targets for clinical treatment of osteosarcoma. Though certainly, more accurate and reliable animal or clinical trials need to be further carried out before lncRNAs can be used in clinical practice.

## MATERIALS AND METHODS

### Literature search strategy

PubMed, Web of Science, Embase, China Knowledge Resource Integrated Database, VIP, Chinese BioMedical and Wan Fang Database were searched by two investigators (M.Y.G. and E.C.Z.) from their initiation date to June 20, 2017. All literatures about lncRNAs for osteosarcoma in the mice were collected, without the limitation of languages using the terms of (long non-coding RNA OR lncRNA) AND osteosarcoma as the search strategy.

### Literature selection and data extraction

Two researchers (S.P.H. and J.L.C.) independently reviewed the titles, abstracts, and full texts and sorted the literatures based on the inclusion criteria. Meanwhile, the data were independently extracted by another two researchers (Y.M.L. and W.Y.W.) according to the characteristics of included studies. All divergences were solved by discussion with a third investigator (Y.P.Y). The following details were extracted for each included study in this meta-analysis: first author name, publication year, characteristics of animals (number, strain, age and gender), animal groups, the methods used to produce osteosarcoma xenograft models (subcutaneous injection or peritoneal metastasis), types of lncRNAs and the measured outcomes (tumor volume or tumor weight).

### Eligibility criteria

#### Types of studies

Any literatures about lncRNAs for osteosarcoma in the mice were collected. All studies that only reported the basic experimental data *in vitro* and clinical cases were excluded.

#### Types of participants

Any strains of mice and osteosarcoma cell lines used to produce osteosarcoma xenograft models were included.

#### Types of interventions

Any intervention methods to correct the altered lncRNA expressions in mouse osteosarcoma models were collected.

### Types of outcome measures

Tumor weight and tumor volume are regarded as the major outcome measures to evaluate the anticancer efficacy by any anticancer therapeutics in preclinical studies. Therefore, in this meta-analysis, any studies that used tumor weight or tumor volume as the outcome measure, regardless the methods used to produce osteosarcoma xenograft models, were collected.

#### Tumor volume

Tumor volume was measured using the formula: tumor volume = 0.5 × a × b (a, the largest diameter of tumor; b, the square of the perpendicular diameter).

#### Tumor weight

At the end of the experiments, osteosarcoma xenografts were detached and weighed as soon as the mice were sacrificed.

### Evaluation of the methodological quality in the individual study

The reporting and design qualities of all included studies in this meta-analysis were evaluated according to STAIR (the initial Stroke Therapy Academic Industry Roundtable), which mainly includes: 1) sample-size calculation; 2) reporting animals excluded from analysis; 3) allocation concealment; 4) randomization; 5) inclusion and exclusion criteria; 6) blinded assessment of outcomes; and 7) reporting potential conflicts of interest and study funding [30]. The methodological qualities of all included studies were assessed by 2 authors (Q.S. and Y.J.W.) and described as a yes or no.

### Statistical analysis

If one outcome was reported by 2 or more studies, data from these studies would be pooled together for reanalysis. In our current meta-analysis, two primary outcomes of tumor volume and tumor weight were individually analyzed. Pair-wise meta-analysis was conducted, for studies directly compared the influence of the rescued lncRNA expression and the control (abnormally expressed lncRNAs) on tumor growth, to verify the pooled relative effects of each intervention for the interest measurement outcome, and the mean differences (MDs) of the post-intervention values from different interventions. As specified by a Cochrane review, we adopted the post-intervention values derived from the baseline values which are comparable between the target lncRNA group and the mimic lncRNA or placebo control group [31].

REVIEW MANAGER 5.1.2 software recommended by the Cochrane Collaboration was applied to analyze the final consequences from the studies to estimate differences between the control and intervention groups. Heterogeneity was evaluated using I^2^, and *p* value under 0.10 by the chi-square (x^2^) test indicates existence of heterogeneity; I^2^ value above 50% indicates existence of a high level heterogeneity among the results. Data from studies with high heterogeneous(I^2^>50) should be pooled for reevaluation by the random-effects model, otherwise, the fixed-effects model was used. When the same outcomes were measured using different instruments across studies, a standardized mean difference (SMD) was used in the meta-analysis to combine the continuous data [31]. All the data included in this meta analysis were the original data extracted from the included papers, and no any data normalization was implemented. When the units used to evaluate the outcomes in different papers were the same, the fixed-effects model was used; otherwise, the random-effects model was used. Funnel plots were established to evaluate the publication bias, when 10 or more studies were included in this meta-analysis.
